# Short-Form Medical Media: A Multi-Platform Analysis of Acne Treatment Information in TikTok Videos, Instagram Reels, and YouTube Shorts

**DOI:** 10.2196/48140

**Published:** 2023-08-09

**Authors:** Christopher J Thang, David Garate, Joseph Thang, Jules B Lipoff, John S Barbieri

**Affiliations:** 1 John Sealy School of Medicine University of Texas Medical Branch Galveston, TX United States; 2 Texas A&M University College Station, TX United States; 3 Department of Dermatology Lewis Katz School of Medicine at Temple University Philadelphia, PA United States; 4 Department of Dermatology Brigham and Women’s Hospital Boston, MA United States

**Keywords:** general dermatology, medical dermatology, acne, acne treatment, social media, TikTok, Instagram Reels, YouTube Shorts, YouTube, Instagram, video, dermatology, skin, patient education, health information, online information, dermatologist

## Introduction

Social media has emerged as a medium for dermatologists to disseminate educational content [1-3]. Adolescents extensively use social media as a resource [1], with 75% of adolescent patients with acne reporting they consult social media for treatment information [4]. However, many dermatologic posts are low in educational quality, especially those made by non–board-certified dermatologists [2]. TikTok has emerged as a wide-reaching, short-form video platform used by millions of adolescents and adults worldwide [1,3]. The short-form video structure has since been emulated in Instagram Reels (IGR) and YouTube Shorts (YTS). However, little is known about how the quality of acne treatment content varies across these platforms and how they compare to each other.

## Methods

To assess the content and educational quality of videos on acne treatment across TikTok, IGR, and YTS, we acquired the top 300 videos per platform from TikTok (search: “acne treatment”), IGR (search: “#acnetreatment”), and YTS (search: “#shorts + acne treatment”) on March 9, 2023. Videos were excluded if they were irrelevant to acne treatment, noneducational, duplicate content, not in English, or made unavailable, as well as if they had hidden metrics or the treatment was unspecified. Video metrics and video engagement rate (VER) ([likes + comments per post]/[followers] × 100] were determined [3]. Video creators were stratified by creator type through review of their profile (dermatologist/dermatology practice, nondermatologist physician/medical clinic, layperson, influencer, or other). “Influencer” was defined as a layperson with at least 40,000 followers [3]. “Other” was categorized as having a specific profession or niche related to skin health (eg, skincare company, aesthetician). Two independent reviewers rated videos using the DISCERN Instrument, which allows health care providers to evaluate the quality of consumer health information, and the Global Quality Scale (GQS), which scores each video based on clinical usefulness [5]. Any discrepancies were handled by consensus. Following video exclusion, a multiple regression analysis was performed to evaluate the association between the platform (ie, TikTok, IGR, and YTS) and creator type with DISCERN and GQS scores, controlling for upload date.

## Results

Of the videos analyzed, 32.8% (82/250) were created by dermatologists/dermatology practices, 5.60% (14/250) were by nondermatologist physicians/medical clinics, 27.2% (68/250) were by laypersons, 20.8% (52/250) were by influencers, and 13.6% (34/250) were by others. The average number of views per video was 1,639,969 on TikTok, 689,897 on YTS, and 27,192 on IGR. DISCERN and GQS scores were significantly higher for dermatologists than any other creator type across all platforms (Table 1). Posts from laypersons had a significantly higher VER compared to posts from dermatologists (Table 1). The most common therapies discussed were benzoyl peroxide, salicylic acid, adapalene, and preventative measures. IGR had a higher rate of discussion of complementary and alternative therapies compared to other platforms (Table 2).

Table 2 presents the number of treatment recommendations among those videos. Topical prescription medications included retinoids, antibiotics, antiandrogens, and steroids. Oral hormonal therapy included birth control pills and spironolactone. Procedural treatments included lasers or lights, chemical peels, extraction, and corticosteroid injections. Complementary and alternative therapies included treatments and suggestions that did not fall into any other category and were nonpreventative measures. Preventative measures included optimizing diet, avoiding pore-clogging makeup, managing stress, exercising, and avoiding picking at pimples.

**Table 1:** Analysis of the DISCERN score, the Global Quality Scale (GQS) score, and the video engagement rate (VER).

		DISCERN				GQS				VER		
		Score, mean	Coefficient (95% CI)	*P* value^a^	Score, mean		Coefficient (95% CI)	*P* value	Rate (%), mean		Coefficient (95% CI)	*P* value
**Platform**												
	Instagram Reels (n=64)	36.9	Ref^b^		2.70		Ref		4.3		Ref	
	TikTok (n=112)	40.8	1.99 (–0.19 to 4.17)	.07	2.94		–0.03 (–0.26 to 0.21)	.83	689.4		1025.87 (479 to 1572.75)	*<.001*
	YouTube Shorts (n=74)	42.4	1.49 (–1.27 to 4.25)	.29	3.26		0 (–0.3 to 0.3)	.98	62.4		492.64 (–199.73 to 1185)	.16
**Creator type**												
	Dermatologist/dermatology clinic (n=82)	46.1	Ref		3.88		Ref		80.7		Ref	
	Nondermatologist physician/medical clinic (n=14)	41.6	–4.41 (–8.09 to –0.73)	*.02*	3.00		–0.88 (–1.28 to –0.49)	*<.001*	86.8		149.1 (–774.82 to 1073.01)	.75
	Layperson (n=68)	36.3	–9 (–11.19 to –6.81)	*<.001*	2.41		–1.46 (–1.7 to –1.22)	*<.001*	973		1069.21 (519.91 to 1618.51)	*<.001*
	Influencer (n=52)	37.5	–8.25 (–10.53 to –5.96)	*<.001*	2.60		–1.27 (–1.52 to –1.02)	*<.001*	70.3		–47.59 (–621.53 to 526.35)	.87
	Other (n=34)	38.1	–7.67 (–10.28 to –5.06)	*<.001*	2.50		–1.37 (–1.65 to –1.08)	*<.001*	131		11.22 (–644.61 to 667.06)	.97

^a^Italicized values are significant.

^b^Ref: reference.

**Table 2:** Acne treatments specified across platforms TikTok, Instagram Reels, and YouTube Shorts and creator types.

	TikTok			Instagram Reels			YouTube Shorts	
	Dermatologist/dermatology practice (n=36)	Nondermatologist (n=76)	Dermatologist/dermatology practice (n=10)		Nondermatologist (n=54)	Dermatologist/dermatology practice (n=36)		Nondermatologist (n=38)
Treatments mentioned, N	97	176	16		84	109		67
Benzoyl peroxide, n (%)	18 (19)	22 (13)	2 (13)		3 (4)	20 (18)		8 (12)
Salicylic acid, n (%)	15 (15)	26 (15)	1 (6)		24 (29)	17 (16)		12 (18)
Other topical OTC^a^ treatments, n (%)	21 (22)	53 (30)	4 (25)		41 (49)	19 (17)		24 (36)
Adapalene (OTC), n (%)	17 (18)	8 (5)	2 (13)		0 (0)	14 (13)		1 (1)
Topical prescription medications, n (%)	6 (6)	24 (14)	2 (13)		1 (1)	12 (11)		3 (4)
Oral antibiotics, n (%)	1 (1)	2 (1)	0 (0)		0 (0)	3 (3)		3 (4)
Oral hormonal therapy, n (%)	3 (3)	6 (3)	1 (6)		0 (0)	5 (5)		1 (1)
Isotretinoin, n (%)	7 (7)	5 (3)	0 (0)		0 (0)	6 (6)		1 (1)
Procedural treatments, n (%)	1 (1)	7 (4)	0 (0)		5 (6)	5 (5)		6 (9)
Complementary and alternative therapies, n (%)	0 (0)	8 (5)	3 (19)		6 (7)	0 (0)		2 (3)
Preventative measures, n (%)	8 (8)	15 (9)	1 (6)		4 (5)	8 (7)		6 (9)

^a^OTC: over the counter.

## Discussion

Our study demonstrates that short-form social media platforms predominantly feature dermatology content created by nondermatologists; however, content produced by board-certified dermatologists was of significantly higher quality as evaluated by the DISCERN and GQS scores. Given the popularity of social media among adolescents with acne [1,4], there is an opportunity for more dermatologists to create content in these spaces where patients seek information. Although the rigorous outcome assessment with DISCERN and GQS scores is a strength of this study, given the rapidly evolving nature of social media, it will be important to reassess these findings over time.

Overall, content on these platforms heavily skewed toward over-the-counter (OTC) treatments, which may reflect the types of treatments that those with acne seek out on social media. However, for many with acne, OTC treatments will be insufficient and prescription therapy will be required. Consequently, dermatologists may find an opportunity on social media to better educate the community regarding prescription acne treatments and to correct misconceptions regarding how to approach OTC management of acne. 

## Abbreviations

GQS: Global Quality Scale
IGR: Instagram Reels
OTC: over the counter
VER: video engagement rate
YTS: YouTube Shorts
